# Toward a Topology-Based Therapeutic Design of Membrane Proteins: Validation of NaPi2b Topology in Live Ovarian Cancer Cells

**DOI:** 10.3389/fmolb.2022.895911

**Published:** 2022-07-15

**Authors:** Leisan Bulatova, Daria Savenkova, Alsina Nurgalieva, Daria Reshetnikova, Arina Timonina, Vera Skripova, Mikhail Bogdanov, Ramziya Kiyamova

**Affiliations:** ^1^ Research Laboratory “Biomarker”, Institute of Fundamental Medicine and Biology, Kazan Federal University, Kazan, Russian Federation; ^2^ Department of Biochemistry and Molecular Biology, McGovern Medical School, the University of Texas Health Science Center, Houston, TX, United States

**Keywords:** SLC34A2, NaPi2b, MX35 antigen, topology, ECD, monoclonal antibody, ovarian cancer

## Abstract

NaPi2b is a sodium-dependent phosphate transporter that belongs to the SLC34 family of transporters which is mainly responsible for phosphate homeostasis in humans. Although NaPi2b is widely expressed in normal tissues, its overexpression has been demonstrated in ovarian, lung, and other cancers. A valuable set of antibodies, including L2 (20/3) and MX35, and its humanized versions react strongly with an antigen on the surface of ovarian and other carcinoma cells. Although the topology of NaPi2b was predicted *in silico*, no direct experimental data are available for the orientation of NaPi2b extracellular domains in cancer cells. The presented results of antibody mapping of untagged NaPi2b in live ovarian carcinoma cells OVCAR-4 provide a platform for current and future epitope-based cancer therapies and serological diagnostics.

## Introduction

Membrane proteins represent at least 30% of all currently sequenced genomes and 60% of known drug targets and are therefore of great interest in understanding and treating diseases (Santos et al., 2017). Moreover, a majority of tumor-targeting compounds are directed against cell integral and membrane-bound proteins (Yin and Flynn, 2015). One of these very attractive targets for cancer therapy is the sodium-dependent phosphate transporter 2B (*SLC34A2*, NaPi2b, NaPi-IIb, and NPT2) which is overexpressed in several malignancies, including ovarian carcinomas (Rangel et al., 2003; Gryshkova et al., 2009), lung (Zhang et al., 2017), thyroid (Kim et al., 2010), and colorectal cancers (Liu et al., 2018). The sodium-dependent phosphate transporter NaPi2b belongs to the type II sodium-dependent phosphate transporter family SLC34 which also includes secondary transporters NaPi2a (NaPi-IIa) and NaPi2c (NaPi-IIc) (Levi et al., 2019). Although the main function in the maintenance of phosphate homeostasis in the human body belongs to the most-studied renal transporter NaPi2a, NaPi2b is involved in maintaining phosphate homeostasis by absorbing inorganic phosphates in the small intestine and a restricted number of other organs and tissues (Levi et al., 2019). Although the topology of renal NaPi2a (Lambert et al., 1999, 2000), as well as of the flounder NaPi2b (Kohl et al., 1998) with large extracellular domain/loop (ECD/ECL) and C- and N-termini facing the cytoplasm was predicted and verified by the heterologous expression of FLAG-tagged and untagged templates in *Xenopus laevis* oocytes, it is not known whether the topological arrangement of the largest extracellular domain facing outside and the N- and C-termini facing the cytoplasm is conserved for all the SLC34 proteins, especially in cancer cells. Our preliminary bioinformatics analysis suggested that the protein encoded by the *SLC34A2* gene has at least 8 potential transmembrane domains (TMD), 5 putative intracellular domains, and 4 putative extracellular loops, with both the N- and C-terminal regions probably facing the cytoplasm (Yin et al., 2008). There are four potential disulfide-bond sites at positions 303, 322, 328, and 350 and six possible N-glycosylation sites at locations 295, 308, 313, 321, 335, and 340 in the putative largest extracellular domain of NaPi2b (Yin et al., 2008). The C-terminal domain is also predicted to have a palmitoylation site (McHaffie et al., 2007). The phosphate transporter NaPi2b was identified as an MX35 antigen by screening a phage cDNA library generated from the OVCAR-3 ovarian cancer cell line (Kiyamova R. G. et al., 2008) with MX35 antibodies (Mattes et al., 1987). Antibodies were generated by immunizing mice with a combination of ovarian cancer tumor cells, and recognized NaPi2b on the cell surface of 80–90% of ovarian carcinoma tumors (Mattes et al., 1987; Welshinger et al., 1997). Epitopes for the monoclonal antibodies (mAbs) MX35 and L2 (20/3) (Kiyamova R. et al., 2008) (epitope MX35) mapped originally within the ECD of the NaPi2b transporter in the frame of 311–340 amino acids by Western blot analysis of GST-fused overlapping recombinant truncated proteins were then narrowed down to 324–338 amino acids (aa) (Yin et al., 2008). A great variety of MX35-based therapeutic antibodies targeting the NaPi2b have then been developed including Rebmab200 (Santos et al., 2013), XMT-1535 (Mosher et al., 2019), XMT-1536 (Bodyak et al., 2021), and XMT-1592 (Fessler et al., 2020). Ultimately, almost all available anti-ECDs of NaPi2b antibodies including MX35, L2 (20/3), Rebmab200, XMT-1535, XMT-1536, and XMT-1592 recognize the same epitope of the NaPi2b protein between the 324 and 338 amino acid residues, however, only mAbs generated by Megale et al. are directed against the other part of the ECD of the NaPi2b protein (Megale et al., 2016).

The first evidence of surface localization of the MX35 epitope within NaPi2b extracellular loop has been obtained by the staining of unfixed ascite tumor cells of women with papillary poorly differentiated ovarian adenocarcinoma using monoclonal antibodies MX35 (Mattes et al., 1987). As mentioned previously, only the flounder NaPi2b topology was mapped previously using recombinant fragments with FLAG-tagged epitopes (Kohl et al., 1998) expressed in oocytes. However, the locations of the ECD, N, and C-terminal domains of the transporter expressed endogenously in cancer cells have not been studied experimentally. The N-terminal domains of membrane proteins are involved in ensuring the accuracy of the entire protein’s insertion into the cell membrane rather than the elongation speed ramp at protein translational starts (Charneski and Hurst, 2014) and can contribute to the correct orientation of all subsequent transmembrane domains in the cell according to the model of sequential membrane protein topogenesis (Hartmann et al., 1989; Bogdanov et al., 2014). From the other side, the topology of N-terminal domains can be uncertain due to their unusual orientational dynamics (Seurig et al., 2019) and post-translational and even post-insertional reorientation (Nass et al., 2022). The C-terminal domain of NaPi2b is likely to be involved in signal transduction, intracellular trafficking, and the surface retention due to presence of multiple cysteine residues capable of reversible acylation (Chamberlain and Shipston, 2015), PDZ domain binding function of which is regulated by phosphorylation or allosterically by other binding partners (Gisler et al., 2001) as it was shown for NaPi2a and NaPi2c (NaPi2b protein counterparts) in renal proximal tubule cells (Levi et al., 2019).

A direct experimental determination of the membrane protein topology has been widely undertaken using confocal microscopy and foreign epitope insertion constructs or panels of monoclonal antibodies with known epitopes, where the assessment of their immunoreactivity has been made before and after cell permeabilization (Banerjee and Swaan, 2006; Nasie et al., 2013). However, we continue to favor mapping the topology of untagged proteins in living cells because foreign tags should be used with caution since such tags may affect the topology of adjacent TMDs with weak hydrophobic topological determinants (e.g. marginally hydrophobic), because their intrinsic flexibility makes them prone to various rearrangements (Bogdanov, 2017). Due to these dynamic, structural, and controversial predictive aspects and the fact that the NaPi2b transporter is an excellent target for anticancer therapy and immunodiagnostics, the aim of this work was to investigate the orientation of the aforementioned three domains (N-termini, largest ECD, and C-termini) in cancer cells by a bioinformatics approach and *in vivo* mapping with different mAbs by confocal microscopy. Despite advances in X-ray, and particularly, the development of advanced CryoEM technologies for acquiring high-resolution structures for membrane proteins, the necessity to identify low-resolution organizational information on membrane proteins in a natural membrane still persists. The structural basis for dynamic and transient topologies remains unapproachable by X-ray crystallography and CryoEM since membrane protein crystals and CryoEM structures of membrane proteins are static (Bogdanov et al., 2018). Although the membrane protein topology gives low-resolution structural information, it can serve as a starting point for various biochemical investigations or three-dimensional structure modeling (Bogdanov, 2017). The dynamic aspects of a protein structure as a function of the physiological state of the cell is best probed in whole intact cells and in a native microenvironment. The identification of specific epitopes for therapeutic antibodies continues to be a major challenge in molecular oncology and immunodiagnostics. NaPi2b may comprise of never been considered, established, continuous, and discontinuous epitopes and therefore represents a new family of potential cell surface markers and targets for the immunotherapy of several types of cancers.

## Materials and Methods

### Cell Culture and Monoclonal Antibodies

The human ovarian carcinoma cell line OVCAR-4, which expresses the NaPi2b transporter endogenously, was purchased from Merck Millipore (United States). Cells were cultured in an RPMI-1640 medium (Paneco, Russia) containing 10% fetal bovine serum, 4 mM l-glutamine, 100 U/mL penicillin, 100 μg/ml streptomycin at 37°C in an atmosphere of 5% CO_2_.

Mouse mAbs against ECD of NaPi2b L2 (20/3) (Kiyamova R. et al., 2008), N-terminal domain of NaPi2b N-NaPi2b (15/1) (Gryshkova V. et al., 2011), and rabbit mAbs against C-terminal domain of NaPi2b (D3V3I, Cell Signaling, United States) were used as the primary antibodies. The goat anti-mouse IgG antibody with Alexa Fluor 488 (Thermo Fisher, United States) and the goat anti-rabbit IgG antibody with Alexa Fluor 488 (Thermo Fisher, United States) were used as secondary antibodies against the corresponding target in this indirect immunostaining approach.

### Bioinformatics Analysis

The CCTOP server was used to predict the number of transmembrane domains in NaPi2b and the topology of its domains. This server allows many programs to create the NaPi2b topology, including HMMTOP, Memsat, Octopus, Phillius, Phobius, Pro, Provid, Scampi, ScampiMsa, and TMHMM (Dobson et al., 2015).

### Confocal Microscopy

OVCAR-4 cells were used to visualize the ECD, N-terminal, and C-terminal domains of NaPi2b. Prior to the experiment, the cells were seeded onto glass bottom dishes (Mattek, United States). The cells were used alive or after being pre-fixed in 4% paraformaldehyde for 15 min and permeabilized in phosphate-buffered saline (PBS) containing 3% bovine serum albumin (BSA) and 0.1% Triton X-100 for 30 min. Cells were incubated with primary antibodies for 1 h at room temperature. Next, the cells were incubated with secondary antibodies for 1 h at room temperature. The nuclei were stained with Hoechst 33342 (Thermo Fisher Scientific, United States) and propidium iodide (PI) (Thermo Fisher Scientific, United States). Staining with PI served to control the membrane integrity of live cells. The fluorescent signal was detected using Z-stack mode on a Zeiss LSM 780 laser confocal microscope (Carl Zeiss AG, Germany). The provided Z-stacks were done using average intensity signals. The thickness of each section was 1 µm. The number of sections (the sample height) varied depending on the conditions of the sample preparation and ranged from 11 to 20 sections. The results were obtained using the Zen Analysis software (Carl Zeiss AG, Germany). The images were collected under the same conditions. Data are representative of multiple independent experiments. Particularly, up to 20 cells were analyzed for each image in three biological repeats.

## Results

### Bioinformatics Analysis of NaPi2b Domain Orientation and Prediction of Its Transmembrane Regions

The CCTOP server (Dobson et al., 2015) was used to predict the topology of the largest ECD (ECL), N-terminal, and C-terminal domains of the NaPi2b in mammalian cells (Supplementary Table S1). Seven out of ten programs on the CCTOP server predicted extracellular localization for the largest ECD of NaPi2b. Seven out of ten programs predicted intracellular orientation of the N-terminal domain and six out of ten algorithms predicted the intracellular location for the C-terminal domain. The number of predicted transmembrane (TM) domains ranges from eight to thirteen membrane-spanning regions with N- and C-terminal domains potentially facing either the cytoplasm or outside. Since fine-tuning of the topological organization of hNaPi2b, including the orientation of re-entrant domains with serine rich QSSS stretches located centrally at the vertex of these mini loops containing Na-Pi-Na binding sites (Patti et al., 2016; Fenollar-Ferrer and Forrest, 2019) was out of scope, the predicted topological organization describes the only way a polypeptide chain is arranged in the membrane, i.e., the number of TMDs and their orientation in the membrane indicates the further verified sidedness of the three largest ECDs (N-termini, ECD, and C-termini). Although the common basic architectural principle of the structure of polytopic membrane proteins is the membrane topology, i.e., the number of TMDs and their orientation in the membrane which subsequently determines the orientation of ECDs, the number of predicted TMDs is very often different and uncertain due to presence of domains with insufficient hydrophobicity (Zhao and London, 2006) which are either underpredicted or not predicted as TMDs. Therefore, predicted entire NaPi2b topology lacks strong preference toward one or the other orientation of either the N-terminal or C-terminal domains or the total number of predicted TMDs. The N- and C-termini can end up either in the cytoplasm or in the extracellular environment. Statistically, if the number of TM segments is even, the N- and C-termini are localized on the same side of the membrane, if the number of TM segments is odd, the N- and C- termini face different sides (Bogdanov et al., 2014). Due to this uncertainty of prediction, an experimental validation of predicted orientation is required. The length of the largest ECD appears to vary from 117 to 128 amino acids within the polypeptide sequence located between positions 236 and 369 of the transporter polypeptide (Supplementary Table S1). It should be noted that the largest extracellular loop boundaries of NaPi2b were proposed to lie between 188 and 361 amino acid residues (Yin et al., 2008). The length of the N-terminal domain varies from 91 to 104 amino acids, and the length of the C-terminal domain varies to a greater extent (58–118 amino acid residues) (Supplementary Table S1). If the length of the C-terminal domain exceeds 100 amino acid residues according to the 6 programs, the C-terminal domain is orientated exclusively towards the cytoplasm (Supplementary Table S1). It can be at least partially supported by the identification of the putative palmitoylation site in the polycysteine region of the C-terminus adjacent to the hydrophobic amino acid stretch predicted to be the last TMD (618–645 amino acid residues) (Lituiev and Kiyamova, 2010). As a result, the effective hydrophobic length of this TMD is increased to trigger a positive mismatch within the membrane (Charollais and Goot, 2009) which usually favors the retention of the C-terminus in the cytoplasm (Dowhan and Bogdanov, 2009). The cysteine string in NaPi2b has been found to alter the sorting, possibly by limiting the basolateral delivery (McHaffie et al., 2007). Furthermore, cysteine-rich palmitoylation motif could stabilize and anchor NaPi2b in biological membranes, as was shown for PLSCR1 (Posada et al., 2014; Herate et al., 2016).

### Analysis of the Largest ECD, N- Terminal, and C- Terminal Domain Topologies of NaPi2b in Ovarian Cancer Cells by Confocal Microscopy

**FIGURE 1:**
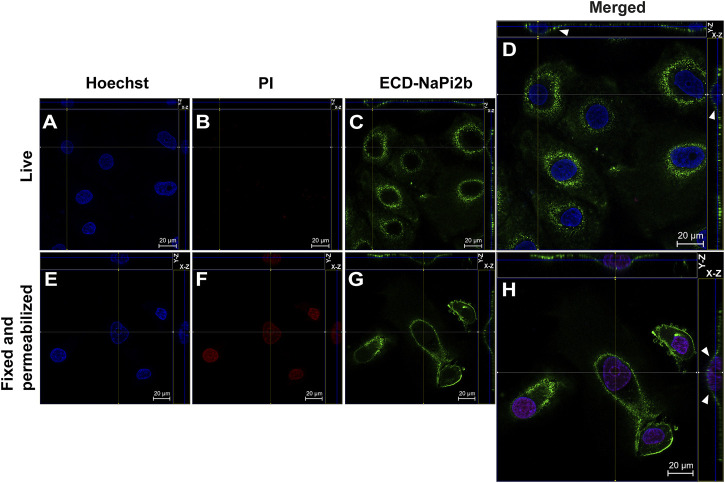
Confocal microscopy analysis of the largest extracellular domain of NaPi2b in ovarian carcinomas cells OVCAR-4 that were either alive **(A–D)** or fixed and permeabilized **(E–H)** and imaged in the Z-stacking mode. **(A,E)** Blue channel (Hoechst); **(B,F)** Red channel (PI); and **(C,G)** Green channel (Alexa Fluor 488), ECD-NaPi2b–mAbs L2 (20/3) directed to ECD of NaPi2b; **(D,H)** Merged images; On the top (*Y*-axis and *Z*-axis combined) and right (*X*-axis and *Z*-axis combined) of each picture, a composite image comprising of numerous photographs obtained at various focus distances is shown. White arrows indicate the position of NaPi2b on the cell membrane.

To determine the orientation of the largest ECD, N-, and C- terminal domains of NaPi2b in OVCAR-4 cells, the mAbs L2 (20/3) directed against the aforementioned ECD (Kiyamova R. et al., 2008), antibody N-NaPi2b (15/1) (Gryshkova V. et al., 2011), and mAbs (D3V3I, cell signaling) directed against the transporter’s N-terminal and C-terminal domains, respectively, were used strategically in confocal microscopy experiments. To ensure that the observed immunofluorescence was entirely a result of the binding of antibodies to the outer leaflet of the OVCAR-4 plasma membrane, we routinely monitored cell integrity during antibody labeling by co-incubating cells with a cell membrane-impermeable probe, propidium iodide (PI). Live cells non-permeable to PI should be impermeable to Abs as well (Zhao et al., 2012). Orientation of the largest predicted ECD of NaPi2b in OVCAR-4 cells is shown in Figure 1.

The monoclonal antibodies L2 (20/3) detect the ECD of NaPi2b in live OVCAR-4 cells without using any reagents that disrupt membrane integrity (native conditions) (Figure 1D), as well as in cells that have been fixed with paraformaldehyde and permeabilized with Triton X-100 (Figure 1H). The Z-stacking (focus stacking) mode combines multiple images taken at different focal distances to provide a composite image with a greater depth of field and therefore enabled us to overlay maximum fluorescent intensities of the membrane over the cytoplasm and justify that the fluorescent signal predominantly derived from the cell membrane plane and therefore confidently confirm the membrane localization of NaPi2b. Live OVCAR-4 cells show the staining of the membrane with L2 (20/3) mAbs (Figures 1C, D) and no nuclear staining with PI confidently demonstrating the extracellular location of domain.

**FIGURE 2:**
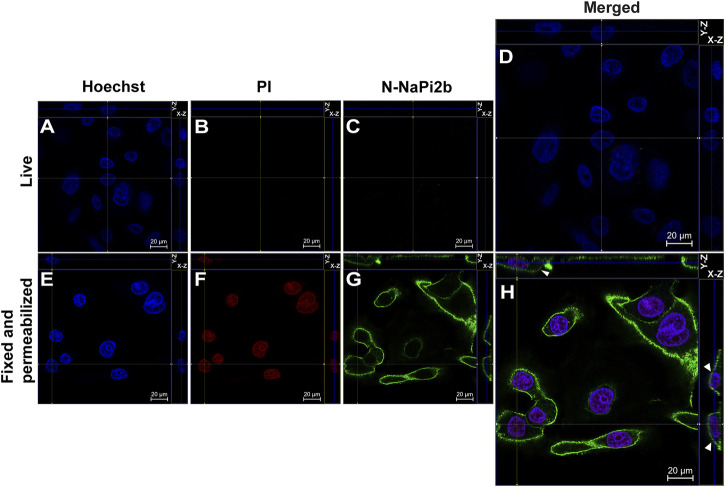
Confocal microscopy analysis of the N-terminal domain of NaPi2b in ovarian carcinomas cells OVCAR-4 that were either intact **(A–D)** or fixed and permeabilized **(E–H)** and imaged in the Z-stacking mode. **(A,E)** Blue channel (Hoechst); **(B,F)** Red channel (PI); and **(C,G)** Green channel (Alexa Fluor 488), N-NaPi2b–mAbs N-NaPi2b (15/1) directed to the N-terminal domain of NaPi2b; **(D,H)** Merged images; On the top (*Y*-axis and *Z*-axis combined) and right (*X*-axis and *Z*-axis combined) of each picture, a composite image comprising of numerous photographs obtained at various focus distances is shown. White arrows indicate the position of NaPi2b on the cell membrane.

Membrane orientation of the N-terminal domain of the NaPi2b is estimated from the results shown in Figure 2. Live and intact OVCAR-4 cells showed no staining with mAbs (15/1) directed against the NaPi2b N-termini and no nuclear staining with PI (Figures 2B, C), whereas, fixed cells treated with a detergent to disrupt the plasma membrane showed both staining patterns (Figures 2F, G). Thus, monoclonal antibodies (15/1) recognize the N-terminal domain of the NaPi2b phosphate transporter exclusively in fixed and permeabilized cells (Figure 2H), but not in live cells (Figure 2D) confidently demonstrating that the N-terminal domain of NaPi2b is located intracellularly. The membrane localization of the NaPi2b was monitored due to fluorescence of Alexa Fluor 488 excited with the 488 nm laser (Figure 2H (X-Z), H (Y-Z) in this indirect immunostaining approach.

**FIGURE 3:**
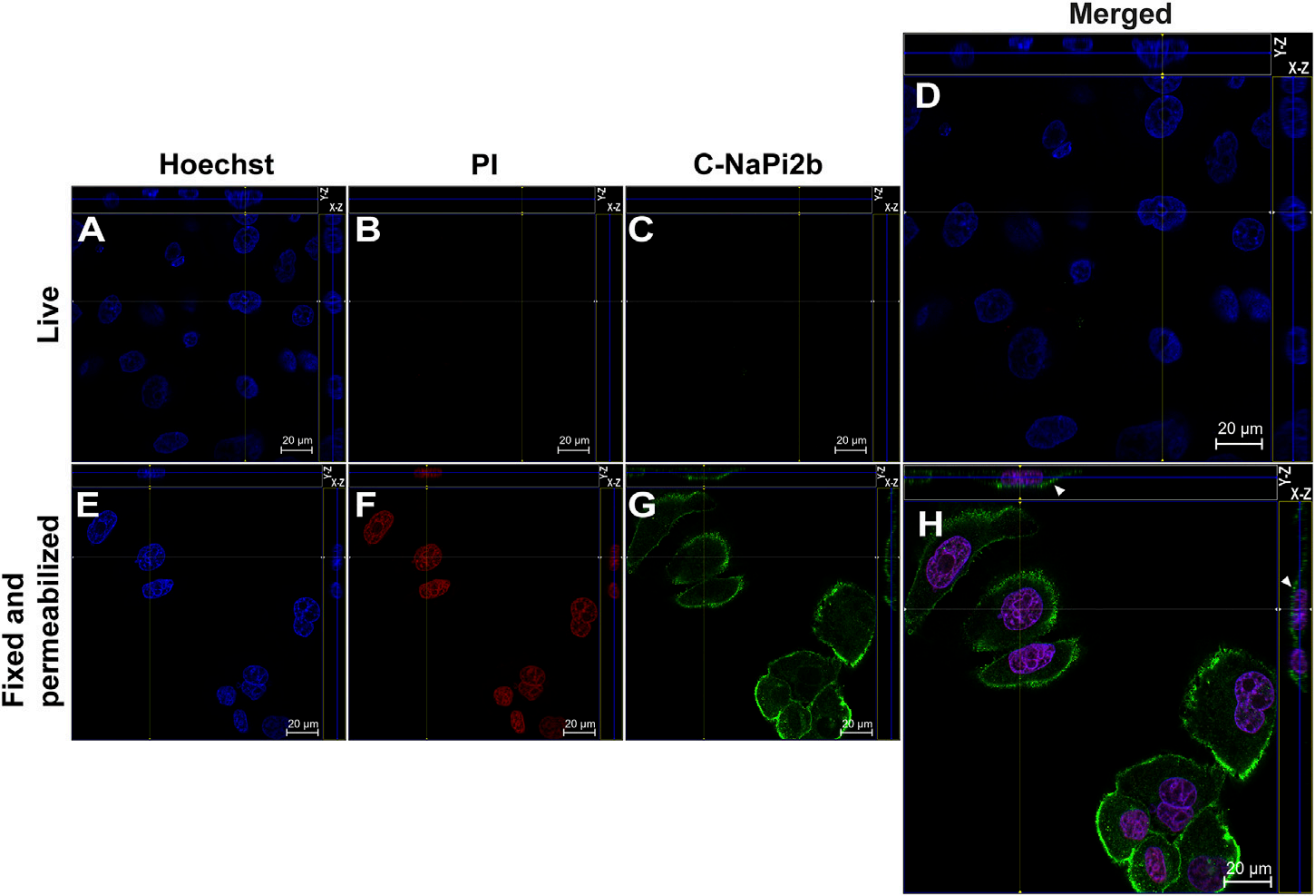
Confocal microscopy analysis of the C-terminal domain of NaPi2b in ovarian carcinomas cells OVCAR-4 that were either alive **(A–D)** or fixed and permeabilized **(E–H)** and imaged in the Z-stacking mode. **(A,E)** Blue channel (Hoechst); **(B,F)** Red channel (PI); and **(C,G)** Green channel (Alexa Fluor 488), C-NaPi2b–mAbs D3V3I (Cell Signaling, United States) directed to the C-terminal domain of NaPi2b; **(D,H)** Merged Images; On the top (*Y*-axis and *Z*-axis combined) and right (*X*-axis and *Z*-axis combined) of each image, a composite image comprising of numerous photographs obtained at various focus distances is shown. White arrows indicate the position of NaPi2b on the cell membrane.

Membrane orientation of the C-terminal domain of NaPi2b is estimated from the results shown in Figure 3. Site-directed anti-C-NaPi2b/SLC34A2 mAbs (D3V3I, cell signaling) detect the C-terminal domain of the NaPi2b phosphate transporter only in cells that are fixed and permeabilized (Figure 3H), but not in live PI-negative OVCAR-4 cells (Figures 3A–D). Permeabilizing the cells with Triton X-100 essentially made all cells positive, not only with PI, but also with these anti-C-termini antibodies, because the antibodies were then able to access the cytoplasmically oriented domain confidently confirming its intracellular orientation. The Alexa Fluor 488 that is excited with the 488 nm laser seen in the Z-stacking mode (Figure 3H (X-Z), H (Y-Z) (indicated by arrows) again confirms the membrane residence of NaPi2b.

This is the first report of the topological structure of NaPi2b obtained by confocal microscopy of live ovarian cancer cells with the set of available antibodies. The data obtained clearly indicate that the N-terminal and the C-terminal domains of NaPi2b are located inside the cell, and the largest extracellular loop of the transporter NaPi2b is located outside the OVCAR-4 cells. Orientation of the N- and C-termini can contribute to the overall topology of membrane proteins and predict whether the number of TMDs is odd or even (Bogdanov et al., 2014). Since both the N- and C-termini of NaPi2b are intracellularly exposed, NaPi2b must have an even number of TMDs in the plasma membrane. It is interesting to note that only the Octopus program of the CCTOP server predicted experimentally determined topology of the ECD, N-, and C-terminal domains of the NaPi2b transporter (Supplementary Table S1). It is important to mention that the Philius and Phobius programs also predict the even number of transmembrane domains (10) with the N- and C-termini in the cytoplasm, but the largest extracellular domain is predicted to be in the cytoplasm (Supplementary Table S1), which is not consistent with our experimental data. Therefore, the NaPi2b consists of eight transmembrane domains with the largest ECD located outside the cell, with both the N-terminal and the C-terminal domains facing the cytoplasm.

### Scope for Mapping the Immunogenic Regions of NaPi2b With Available Monoclonal Antibodies

Membrane proteins, due to their localization, are attractive targets for targeted therapy of oncological diseases. For the moment, 127 therapeutic mAbs are represented on the market, and 77 of them are directed against membrane proteins (Salazar et al., 2021). Currently, several mAbs against the NaPi2b have been generated, including the humanized antibodies XMT-1536 (https://www.mersana.com/pipeline/xmt-1536/) and XMT-1592 (https://www.mersana.com/pipeline/xmt-1592/), which are undergoing clinical trials for the treatment of ovarian and lung cancers. All these antibodies are MX35-based antibodies and are directed against the epitope MX35 located within the largest extracellular loop of NaPi2b.

**FIGURE 4:**
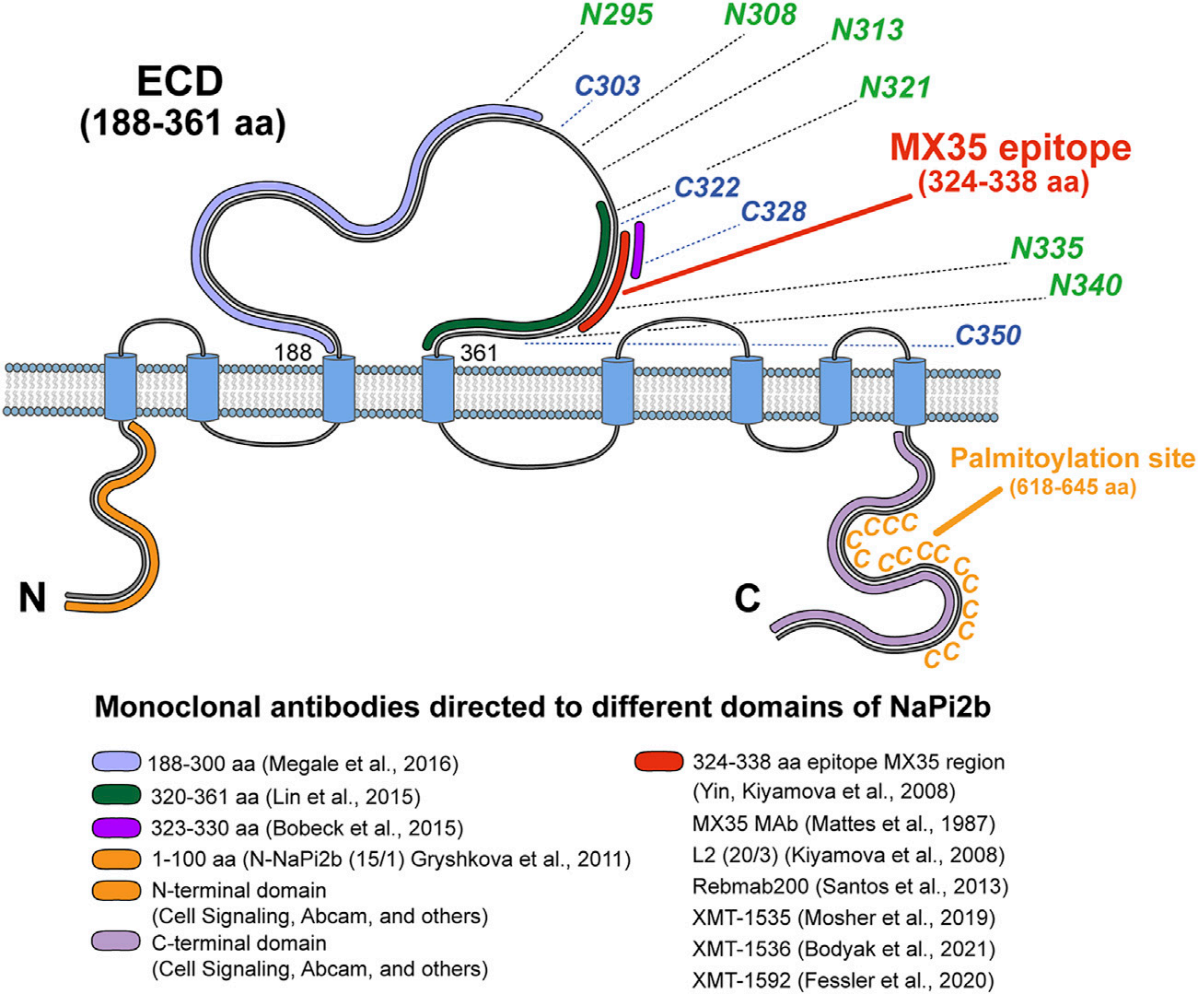
Topological model of NaPi2b with color-coded antigenic regions recognized by available monoclonal analytical and therapeutic antibodies targeting different extramembrane domains. The epitope’s region in ECD of NaPi2b for mAbs, generated by: Megale et al. (2016), 188–300 aa, marked in light purple; Lin et al. (2015), 320–361 aa, marked in green; Bobeck et al. (2015), 323–330 aa, marked in purple; Yin, Kiyamova et al. (2008), Mattes et al. (1987), Kiyamova et al. (2008), Santos et al. (2013), Mosher et al. (2019), Bodyak et al. (2021), Fessler et al. (2020), 324–338 aa, marked in red; Epitope region in N-termini and C-termini of NaPi2b for mAbs, generated by: Gryshkova et al. (2011), Cell Signaling, Abcam, and others against N-terminal domain of NaPi2b, 1–100 aa, marked in orange; Cell Signaling, Abcam, and others against С-terminal domain of NaPi2b, the region is unknown, marked in lavender. Positions of cysteine and asparagine amino acid residues potentially contributing to the oxidative folding and formation of disulfide bonds and N-linked glycosylation of the largest ECD, and palmytoilation site in C-terminal domain are also shown.

We analyzed all available data and provided a scope for the recognition of NaPi2b extracellular domains by all available arsenal of therapeutic and analytical Abs using the schematic model of human NaPi2b (Figure 4). The model includes positions of cysteines potentially engaged in disulfide bonds, putative asparagine-linked N-glycosylation sites, the location of the MX35 epitope, and the palmitoylation sites in the largest ECD, and the C-terminal domain of NaPi2b, respectively (Figure 4). Provided color-coded scheme rationalizes at first time antigenic regions within largest NaPi2b ECD recognized by available monoclonal antibodies. Both MX35 and L2 (20/3) are mouse mAbs-specific for the MX35 epitope of human sodium-dependent phosphate transporter NaPi2b. The monoclonal antibodies MX35 were generated from mice immunized with a cocktail of human ovarian carcinoma cells (Mattes et al., 1987), while L2 (20/3) mAbs were produced in mice immunized with the truncated recombinant NaPi2b protein (ECD, 188–361 aa) (Kiyamova R. et al., 2008). The monoclonal antibodies L2 (20/3) blocks the phosphate-mediated current driven by NaPi2b expressed in renal cancer cells SK-RC-18 (Kiyamova et al., 2011) while binding of MX35 mAbs to a largest ECD of NaPi2b inhibits the uptake of inorganic phosphate in HEK293 cells stably expressing WT protein (Gryshkova V. S. et al., 2011). As an approach toward elucidating the mechanism by which this ECD folds and affects phosphate transport, we have to undertake the definition of the binding sites and the specificity of these and other mAbs shown in Figure 4. Rebmab200, a humanized version of MX35 antibodies (Santos et al., 2013; Lindegren et al., 2015), was further renamed as XMT-1535 (Bodyak et al., 2021). Using dolaflexin-based conjugation technology, an antibody–drug conjugate (ADC) XMT-1536 was developed from XMT-1535 (Bodyak et al., 2021; Yurkovetskiy et al., 2021). Another ADC XMT-1592 was generated from XMT-1535 antibodies using dolasynthen conjugation technology (Fessler et al., 2020). It is important to state that despite the availability of various sets of mAbs directed to different regions of the large extracellular domain of the transporter, therapeutic effectiveness in clinical trials was so far demonstrated for only the mAbs-targeting MX35 epitope particularly in the first-in-human study of humanized XMT-1536 (https://clinicaltrials.gov/ct2/show/NCT03319628) and XMT-1592 (https://clinicaltrials.gov/ct2/show/NCT04396340) in patients with ovarian cancer and non-small cell lung cancer.

## Discussion

The identification of local and gross membrane protein misfoldings and stable structure- and cancer-type-specific epitopes for therapeutic antibodies such as the elucidation of their functions *in vivo* continue to be a major challenge in molecular oncology and immunodiagnostics. Exactly how this structural plasticity and dynamics of EMDs comprising such epitopes are achieved and controlled has not been clear. It should be noted that the topology of heterologously expressed renal NaPi2a was investigated predominantly in *Xenopus laevis* oocytes by different transmembrane mapping techniques including the immunohistochemical approach and N-glycosylation topology mapping with engineered glycosylation tags as topological probes (Lambert et al., 1999)). Although polyclonal anti-peptide antibodies against the N- and C-termini of NaPi2a were independently utilized for topology mapping, the insertion of the FLAG epitope (DYKDDDDK) location could affect the topology of NaPi2a due to the presence of five negatively charged residues in their sequences. The location of ECD, N-, and C-terminal domains of the NaPi2b transporter has been studied experimentally only on flounder NaPi2b recombinant fragments with FLAG-tagged epitopes (Kohl et al., 1998). In 2008, a topological model of NaPi2b consisting of at least eight potential transmembrane domains (TMD), five putative intracellular domains, and four putative extracellular loops, with both N- and C-terminal regions probably facing the cytoplasm in ovarian cancer cells was computationally predicted (Yin et al., 2008). Since cysteine and aspartate residues located in different extramembrane domains of NaPi2b (ECD and C-terminus) are functionally important, the Abs mapping approach provides a reasonable and perhaps the only one possible alternative to the substituted cysteine accessibility method (SCAM™) and N-glycosylation mapping technique (Bogdanov, 2017). Abs mapping represents one of the best available approaches so far for systematic topology mapping and topological analysis of untagged NaPi2b and other SCL34 proteins in live cancer cells.

Our results provide the first experimental evidence for the intracellular location of the N- and C-termini, and the extracellular location of the largest extracellular domain (loop) of the untagged NaPi2b transporter localized primarily in the plasma membrane, as demonstrated by confocal microscopy in cancer cells. Our model is consistent with the model of NaPi2b predicted by homology modeling (Patti et al., 2016) despite the limitations described below and the 3D structures of NaPi2b predicted computationally from the protein sequence with atomic accuracy by AlphaFold2. Correspondingly to the NaPi2a model (Fenollar-Ferrer et al., 2014), an outward and inward facing homology model of the Na-coupled phosphate co-transporter NaPi-IIb from flounder was generated using a repeat-swapped VcINDY model (Patti et al., 2016) and the modeled structure of hNaPi-IIa (Fenollar-Ferrer and Forrest, 2019) as templates. However, this model is missing its largest extracellular loop (ECD/ECL) connecting TMDIII and TMDIV, as well as the last two TMDs. These TMDs and ECD were intentionally omitted from the final homology model for “functional” reasons since no equivalent templates in VcINDY was respectively found suggesting that these TMDs are not part of the transport core fold despite the fact that a large extracellular loop can be associated with the transport function and postulated conformational changes (Patti et al., 2016). Nevertheless, Na1-site perturbing mutations were successfully mapped onto this homology model and supported by the positioning of probes to examine transport dynamics and kinetics (Patti et al., 2016). The NaPi2b model for discrimination of continuous epitopes’ regions mapped with various mAbs, including MX35 antibodies directed against different NaPi2b ECDs is presented on Figure 4, including cysteine residues potentially engaged in disulfide bonds, asparagine-linked N-glycosylation sites, the MX35 epitope, and the palmitoylation site within the NaPi2b C-terminal domain. We recently demonstrated that MX35 antibodies as well as L2 (20/3) antibodies recognize conformationally exposed epitope MX35 structurally constrained by disulfide bonds and carbohydrates attached at asparagine-linked glycosylation sites which can potentially contribute to the foldability of the largest ECD containing this epitope (Kiyamova et al., 2020; Bulatova et al., 2021).

Whether an epitope is conformationally constrained by disulfide bonds and whether N-glycosylation stabilizes structurally tethered conformation of the epitope is still unknown. Obviously, the epitope will be partially misfolded or folded differently if the disulfide bonds are unformed or mismatched. We suppose that an aberrant folding and/or transmembrane misassembly may determine bioavailability (immunogenicity) of this epitope and therefore “druggability” of membrane proteins as a target in cancer cells. We postulate that epitope MX35 recognition depends on its unique conformation specific only to cancer cells making it a potential cancer-specific epitope during co-translational oxidative folding and N-glycosylation events in cancer cells (Kiyamova et al., 2020; Bulatova et al., 2021). This assumption is supported by the fact that even though the transporter NaPi2b is expressed in several normal tissues, the MX35 mAbs are accumulated predominantly in cancer cells, as demonstrated during the first clinical trials involving the pharmacokinetics, biodistribution, and intraoperative radioimmunodetection of radiolabeled MX35 in patients with advanced epithelial ovarian cancer (Rubin et al., 1993). Digital images confirmed the specific uptake of radiolabeled mAbs MX35 F (ab’) in tumor cell foci rather than the adjacent non-tumor tissues (Finstad et al., 1997). Intraperitoneal administration of 211At-MX35 F(ab9)2 achieves therapeutic absorbed doses in microscopic tumor clusters without significant toxicity indicating that antibodies do not accumulate in normal tissues (Andersson et al., 2009). Specific accumulation of MX35 mAbs in cancer cells may be explained by its overexpression in ovarian cancer cells (Gryshkova et al., 2009), but we cannot exclude the fact that MX35 antibodies and their humanized versions recognize the potential “cancer-specific” epitope of the transporter due to conformation provided only by cancer cells. This hypothesis is the subject of our current independent investigation.

Interestingly, recent attempts to generate antibodies to epitopes other than the MX35 epitope led to the production of several antibodies directed toward the largest ECD. These mAbs include those generated recently by Genentech derived from the sequence corresponding to the region between 320 and 361 amino acids of full-length human NaPi2b (Lin et al., 2015) and antibodies directed against synthetic peptides corresponding to an amino acid sequence of 188–300 residues in the non-overlapping portion of the NaPi2b protein epitope for MX35 Abs (Megale et al., 2016). Egg-yolk anti-h16 antibodies deposited in the avian egg and directed against the TSPSLCWT sequence (323–330 aa) within epitope MX35 of NaPi2b also were produced (Bobeck et al., 2015). It was shown that monomethyl auristatin E (MMAE) conjugated antibodies generated by Genentech inhibited tumor growth and caused tumor regression in xenograft animal tumor models, OVCAR-3-X2.1, and IGROV-1 derived from human ovarian cancer and from human non-small cell lung adenocarcinoma epithelial cells, respectively (Lin et al., 2015). Even though the antibodies generated by Genentech were evaluated in pre-clinical studies as an effective new therapy for the treatment of NSCLC and ovarian cancer, no additional information is available about the clinical studies of these antibodies. To date, only mAbs XMT-1536 (upifitamab rilsodotin (UpRi)) and XMT-1592 are under investigation in clinical trials for ovarian and lung cancers treatment in accordance with the www.clinicaltrials.gov server. UpRi, a first-in-class ADC-targeting NaPi2b, utilizes the Dolaflexin platform to deliver about 10 DolaLock payload molecules conjugated to auristatin (anti-tubulin agent) per antibody (Yurkovetskiy et al., 2021). UpRi is being studied in UPLIFT, a single-arm registration study in patients with platinum-resistant ovarian cancer (NCT03319628). In July 2021, UPGRADE, a Phase 1 umbrella study, combining UpRi with other ovarian cancer therapies in patients with platinum-sensitive high-grade serous ovarian cancer (NCT04907968), was initiated. Antibodies XMT-1592, based on the dolasynthen platform, are in phase 1 dose escalation trial (NCT04396340) in patients with tumors likely to express NaPi2b. Thus, NaPi2b represents a promising target for antitumor therapy due to its predominant membrane localization, existence of potential cancer-specific epitope, and increased expression in several tumors. However, we have shown recently that neo-adjuvant therapy with carboplatin and paclitaxel reduces the protein expression of the NaPi2b transporter in tumors of ovarian carcinoma, which calls into question the use of correspondent antibodies for ovarian cancer patients’ treatment (Nurgalieva et al., 2021).

Site-directed antibodies’ transmembrane topology mapping of untagged NaPi2b in live and intact ovarian cancer cells allowed us to experimentally validate a previously predicted model for the transmembrane organization of extramembrane domains (Yin et al., 2008) and provide an up-to date experimental platform for NaPi2b epitope-based cancer immunodiagnostics and immunotherapy. The establishment of the sidedness of surface-hidden N-terminal and C-terminal domains and demarcation of the robustness of the topology of NaPi2b in a normoxic condition is critically important for current and future studies aimed at understanding the roles of defined regions in function, folding, and antigenicity as well as the development of a new immunotherapeutic approaches.

An effective design of therapeutic monoclonal antibodies is dependent on understanding the membrane protein structure and the rules that govern the folding and topogenesis of native membrane proteins in healthy and cancer cells. These structural features appear to be crucial factors for the development of recombinant potent therapeutic monoclonal antibodies acting by specifically binding an extracellular epitope on the surface of a membrane protein with high affinity. Therefore, an actual hallmark of the membrane protein’s “druggability” with mAbs is the requirement for the extent of solvent exposure of folded extramembrane domains making it fully accessible to therapeutic antibodies. This view challenges the dogma that a solvent-accessible active site of an enzyme is only the “Achilles’ Heel” of membrane proteins which should serve as the primary target for different drugs.

## Data Availability

The original contributions presented in the study are included in the article/Supplementary Material; further inquiries can be directed to the corresponding authors.
