# Supplementary material for: Toward a Topology-Based Therapeutic Design of Membrane Proteins: Validation of NaPi2b Topology in Live Ovarian Cancer Cells
Source: Front Mol Biosci. 2022 Jul 15;9:895911. doi: 10.3389/fmolb.2022.895911 (PMC9335355; doi:10.3389/fmolb.2022.895911)
Supplement: Supplementary file 1 [file Table1.DOCX]

Supplementary Material

**Supplementary Table 1.** Bioinformatic analysis of sodium-dependent phosphate transporter NaPi2b domains topology using the programs of CCTOP server

|  | **Program** | **Number of**  **transmembrane**  **domains** | **N-terminal**  **domain**  **region, aa** | **Length,**  **aa** | **Topo-**  **logy** | **The large Extracellular domain (ECD), aa** | **Length,**  **aa** | **Topo-**  **logy** | **C-terminal domain region, aa** | **Length,**  **aa** | **Topo-**  **logy** |
| --- | --- | --- | --- | --- | --- | --- | --- | --- | --- | --- | --- |
| 1 | HMMTOP | 13 | 1-100 | 100 | C | 235-362 | 128 | E | 614-690 | 77 | E |
| 2 | Memsat | 9 | 1-91 | 91 | C | 236-359 | 124 | E | 632-690 | 59 | E |
| 3 | Octopus | 8 | 1-96 | 96 | C | 239-362 | 124 | E | 574-690 | 117 | C |
| 4 | Philius | 10 | 1-97 | 97 | C | 242-361 | 120 | C | 576-690 | 115 | C |
| 5 | Phobius | 10 | 1-100 | 100 | C | 242-361 | 120 | C | 576-690 | 115 | C |
| 6 | Pro | 11 | 1-104 | 104 | E | 240-366 | 127 | E | 573-690 | 118 | C |
| 7 | Prodiv | 11 | 1-97 | 97 | E | 242-361 | 120 | E | 573-690 | 118 | C |
| 8 | Scampi | 11 | 1-91 | 91 | C | 241-360 | 120 | C | 633-690 | 58 | E |
| 9 | ScampiMsa | 11 | 1-92 | 92 | C | 241-363 | 123 | E | 633-690 | 58 | E |
| 10 | TMHMM | 11 | 1-102 | 102 | E | 245-361 | 117 | E | 577-690 | 114 | C |

^*C – cytoplasmic/intracellular orientation, E – extracellular orientation, aa – amino acids^
